# Influence of Posterior Corneal Surface Irregularities on the Attachment of an Artificial Endothelial Layer (EndoART)

**DOI:** 10.3390/jcm14103395

**Published:** 2025-05-13

**Authors:** Ruth Donner, Michal Klimek, Gerald Schmidinger

**Affiliations:** Department of Ophthalmology and Optometry, Medical University of Vienna, Spitalgasse 23, 1090 Vienna, Austria; n12117073@students.meduniwien.ac.at (M.K.); gerald.schmidinger@meduniwien.ac.at (G.S.)

**Keywords:** cornea, endothelium, artificial graft

## Abstract

**Purpose:** This study aimed to refine the criteria for EndoART implantation regarding posterior corneal irregularity; to improve the selection of candidates for this synthetic alternative to endothelial keratoplasty. **Methods:** This study analyzed the impact of posterior corneal surface elevation differences; anterior chamber depth (ACD); and preoperative corneal pachymetry on the success of EndoART implant adhesion. Patients undergoing EndoART implantation at the Medical University of Vienna were assessed using OCT to measure corneal irregularities. Postoperative outcomes, including re-bubbling rates; implant adhesion; and visual acuity changes, were monitored. **Results:** EndoART successfully adhered in eyes with moderate posterior irregularities (elevation differences up to 204 µm). Severe irregularities (elevation differences > 200 µm) resulted in implant detachment. No significant correlation was found between corneal pachymetry or ACD and adhesion failure. Glaucoma devices and prior penetrating keratoplasty did not significantly affect adhesion. Some cases required re-bubbling, and patients reported pain reduction and moderate improvements in visual acuity. **Conclusions:** This study found that EndoART implantation can be successful despite posterior corneal irregularity. EndoART represents a viable solution for patients with poor biological graft survival prognosis, including those with glaucoma or prior surgeries, expanding its potential use and addressing the global donor cornea shortage.

## 1. Introduction

Over the past decade, there has been a growing preference for lamellar corneal grafting procedures that require less transplanted tissue. In the United States, the frequency in endothelial transplantation techniques (EK) overtook penetrating keratoplasty for the first time in 2012, and a report by the German Keratoplasty Registry shows that posterior lamellar transplantations accounted for approximately 60% of all keratoplasties performed in Germany in 2016 [1,2]. Of these, 92% were Descemet membrane endothelial keratoplasties (DMEKs) [1,2]. The success rates of primary EK are high, ranging from 83% to 96%, 5 years after DMEK [3]. DMEK is favored over penetrating keratoplasty for endothelial dysfunction due to advantages regarding minimized invasiveness, higher safety profile and faster visual recovery and is currently considered to be the gold standard of treatment for patients with corneal edema due to endothelial failure [4].

Indications for DMEK can result from several primary (e.g., Fuchs dystrophy) or secondary endothelial failure (e.g., pseudophakic bullous keratopathy, graft failure after penetrating or endothelial keratoplasty, or endothelial decompensation after glaucoma surgery) or from damage due to high intraocular pressure (IOP) [2,5].

Despite its benefits, DMEK, like all biological corneal grafts, is associated with a number of possible postoperative complications such as graft failure, immunological graft rejection, or keratitis, which can compromise the quality of surgical outcomes [6,7,8].

Furthermore, with a global shortage of available donor corneas, meeting the increasing demand is impossible [9]. A global survey on corneal transplantation and eye banking estimates that 12.7 million patients are waitlisted for corneal donor tissue. This highlights a significant global shortage due to the limited availability of donors. This study further reveals that 53% of the global population cannot access corneal grafts, primarily because of the essential infrastructure requirements for eye banking and the need for specialized surgical expertise [2,9].

To overcome this shortage, efforts have been made to create medical devices substituting biological tissue. EndoArt (EndoArt, Eye Yon Medical, Israel) was designed to act as an artificial replacement for an endothelial graft. It is made of acrylic hydrophilic flexible material (copolymer of hydroxyethyl methacrylate and methyl methacrylate), measures 50 μm in thickness and 6.5 mm in diameter and is shaped to follow a standard corneal posterior curvature (radius 6.8 mm) with the goal of acting as a fluid barrier to replace and mimic the function of the corneal endothelium [2,4]. Similarly to biological endothelial grafts, the device adheres to the inner corneal surface. Once in place, it prevents aqueous penetration into the central corneal stroma, resulting in a reduction in stromal edema and improvement in corneal transparency.

Irregularities of the posterior corneal surface can impair implant adherence, potentially affecting surgical outcomes and patient recovery [4,10]. In this study, preoperative criteria, including factors such as corneal surface morphology, patient history and surgical preparation techniques, were reviewed and analyzed to determine their correlation with postoperative results. By examining these criteria, this study aimed to identify key predictors of successful graft adhesion and optimize the selection and preparation process for improved outcomes in DMEK procedures.

## 2. Methods

Patients eligible for implantation of an artificial endothelial replacement were recruited from the cornea outpatient service at the Medical University of Vienna. All patients had undergone multiple prior surgeries and had experienced endothelial dysfunction. Eyes that were eligible for implantation of an artificial endothelial layer showed no severe scarring or phthisis.

After obtaining informed consent, patients were scheduled for EndoArt (EndoArt, Eye Yon Medical, Israel) implantation. Surgery was performed according to the standard procedure as recommended by the manufacturer and prior experiences by other implanting surgeons, replacing previous biological grafts (DMEK and DSAEK (Descemet stripping automated endothelial keratoplasty)) with the implant and placing a full-thickness anchoring suture and an 18 mm bandage contact lens on the eye [2]. The gas used for all surgery was 10% C3F8. The postoperative topical regimen included an antibiotic eye drop (ofloxacin) 4 times daily for 1 week and a corticosteroid eye drop (dexamethasone) 5 times daily as a longer-term treatment. Further immunosuppressive treatment was not necessary due to the synthetic composition of the implant. Early follow-up visits were performed at 3 h, 1 day, 1 week, 1 month and 3 months. Re-bubbling was performed as needed with a lack of adhesion after resorption of the gas bubble. Follow-up visits included IOP measurements, anterior-segment optical coherence tomography (Casia 2, Tomey) and slit lamp examination. All patients were instructed to remain in a strictly supine position at least until the 1-week follow-up appointment.

### Posterior Corneal Surface

**Figure 1:**
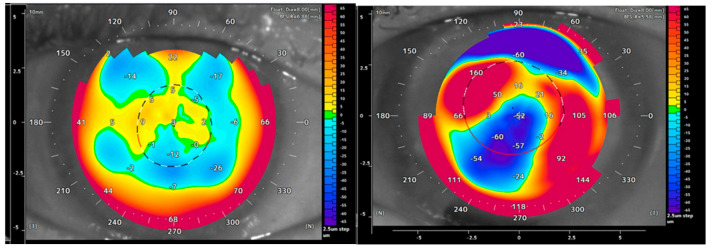
Posterior elevation map displaying a moderately irregular posterior surface (Patient 1 preoperatively; (**left**)) and an irregular corneal curvature pattern (Patient 6 preoperatively; (**right**)). Despite interventions such as re-bubbling and sutures, implant adhesion could not be achieved in Patient 6, while adhesion was immediate in Patient 1.

The evaluation of posterior corneal surface was based on preoperative OCT topography, which offered parameters including average K measurements as well as focal elevation maps and anterior chamber depth. Average K provides a general measure of the mean posterior corneal curvature. This parameter was complemented by the maximum/minimum focal elevation difference compared to a reference sphere, defined by the highest and lowest elevation points on the posterior cornea. This addition was necessary because the standard evaluation of the vertical and horizontal meridians does not always capture the complexity of highly irregular curvature patterns. These irregularities are better represented by the maximum elevation difference, as illustrated in the posterior surface elevation maps (Figure 1).

Patients with glaucoma drainage devices have shown a higher rate of implant detachment, necessitating additional interventions, mainly re-bubbling [11]. The presence of glaucoma devices may disrupt the implant’s adherence due to altered aqueous humor dynamics or mechanical interference. While specific data on the effects of corneal pachymetry on EndoArt adhesion are limited, significant corneal edema or thinning could potentially impact the implant’s ability to adhere properly [11]. Excessive thickness might hinder effective placement, while extreme thinning could compromise structural support. Variations in anterior chamber depth (ACD; measured from the endothelium) could influence implant positioning: an excessively shallow anterior chamber may pose challenges during implantation, increasing the risk of complications. Conversely, a very deep anterior chamber might affect the stability and centration of the implant [12].

## 3. Results

Patients who underwent EndoART implantation presented with diverse surgical histories and comorbidities (Table 1). There were no intraoperative complications, and none of the patients experienced pupillary block or increases in IOP in the early postoperative phase.

Re-bubbling was necessary in 4 of 11 patients, representing a re-bubbling rate of 36%.

Patient 4 required re-bubbling after complete resorption of the initial 10% C3F8 bubble and remained in inpatient care for 3 days in strict supine positioning, after which adhesion was reached and sustained. Patients 3 and 9 showed implant dehiscence that was resistant to all positioning efforts including re-bubbling, supplementary suturing and strict supine positioning. These were also the patients who showed higher irregularity in the posterior corneal curvature than the other patients in this cohort. Implant failure due to a lack of adhesion required further surgical intervention with biological grafts. Patient 8 showed a moderately high degree of posterior corneal irregularity compared to other patients and required a single re-bubbling, which was able to accomplish implant adhesion after 7 days.

Primary adhesion of the implant was observed in eyes with a mean posterior max. elevation difference between 46 µm and 204 µm. Cases with detachment had a mean posterior difference in elevation of 76 µm to 414 µm. The ACD was between 2.29 and 3.93 mm in eyes with immediate implant adhesion. Persistent detachments were noted with an extremely deep anterior chamber (Patient 6) as well as with a very shallow chamber (Patient 9). The preoperative corneal thickness ranged from 533 and 1034 µm in cases with immediate attachment and 559–741 µm in cases with significant detachment (Table 1).

The minimal corneal thickness dropped from an average of 678 µm preoperatively (min 533 µm, max 1034 µm) to 506 µm within the first month of postoperative follow-up (min 381 µm, max 690 µm). While Patient 1 improved from a visual acuity of counting fingers to 0.5 logMAR, the other patients’ visual acuity did not improve by more than one line, or it did improve from realizing hand movements to being able to count fingers. Though these increases in visual acuity were moderate, all patients consistently reported improvements regarding previous photophobia and pain.

Concerning other complications, three patients experienced corneal infections several months after implantation—all resolved with topical therapy. However, all three cases showed corneal thinning and scarring of the stroma after the event.

## 4. Discussion

This analysis of 11 patients produced results that are novel in the literature for EndoART implantation: moderate posterior corneal irregularity (up to a difference in posterior elevation of approximately 200 µm) can allow for successful implant adhesion.

Results showed that primary attachment of the EndoART implant was successful in eyes with a mean posterior irregularity profile within a specific range, while extreme irregularities (>200 µm elevation difference) led to persistent detachment. Preoperative corneal pachymetry varied widely, but no clear correlation with adhesion failure was identified, and dehiscence occurred in very deep as well as very shallow anterior chambers. While some cases required re-bubbling, immediate attachment could be achieved despite moderate posterior curvature irregularity, suggesting some allowance in this regard. Further, it seems that glaucoma filtering surgery as well as glaucoma implants do not necessarily interfere with implant adhesion [11].

In two of the cases (cases 6 and 9) included in this analysis, posterior elevation data showed a highly irregular surface (Figure 1). In these cases, none of the available measures (multiple re-bubblings, placing up to four fixation-sutures, prolonged supine patient positioning) could achieve stable adhesion of the implant for more than 24 h; thus, it appears that these levels of irregularity are too pronounced to allow for successful results. Given that the EndoART implant is thicker and cannot replicate the biomechanical properties of the Descemet membrane/endothelium complex used in DMEK, it may be more susceptible to detachment in eyes with irregularities in the posterior corneal surface. Therefore, in patients with complex posterior surface irregularity, biological grafting might still be the most advisable choice when endothelial replacement is necessary.

Patients with moderate irregularity (up to a difference of 204 µm in the posterior elevation map) showed mixed results. Patients 2 and 3 achieved immediate implant adhesion, while Patient 8 required a single re-bubbling. While other factors may also play a role in the tendency for dehiscence, these cases could point towards a level of posterior irregularity that disables implant adhesion. While the patients in this analysis exhibited fairly similar levels of posterior corneal irregularity, measurements from larger cohorts are likely to help establish a more precise cut-off range beyond which the risk of implant dehiscence becomes too high to permit EndoART implantation.

The prior penetrating keratoplasty, though often associated with irregular posterior corneal curvature, did not necessarily hinder adhesion in this series. However, it does seem likely that implant positioning is crucial for adhesion, as the implant may adhere more easily if not placed at the seam between the donor and recipient cornea. Similar observations and suppositions were formulated in the prior literature after implants had shifted from areas with higher irregularities to areas with a smoother profile [4].

Although the small sample size makes it unclear at what degree of posterior corneal irregularity that the likelihood of implant dehiscence surpasses its chances of adherence, this case series provides valuable insight into the factors influencing implant behavior. While highly irregular posterior surfaces seem to prevent adhesion, moderate irregularities were tolerated. This observation is significant, seeing as the majority of eyes that are currently eligible for EndoART implantation have a multitude of ocular comorbidities as well as multiple prior surgeries, both of which increase the likelihood for corneal curvature irregularities, and posterior corneal irregularity is widely considered to be a contraindication for EndoART implantation.

Patient 4 experienced graft dehiscence despite having a comparatively regular posterior corneal curvature. After inpatient care for three days with re-bubbling, implant adhesion was stable. Although other factors cannot be ruled out as contributing to this case, it appears possible that adherence to supine positioning in the early postoperative phase may have been inadequate in the context of independent home care. This observation stresses the importance of supine positioning and thorough explanation of the gas bubble’s significance to patients and differs from observations after DMEK surgery [13].

Intraocular implants after glaucoma surgery did not appear to have a noteworthy effect on the implant’s ability to adhere and function in the recipient bed. This observation could ease the apprehension expressed in earlier case studies towards EndoART implantation with glaucoma tubes [4]. Although a risk for faster gas loss through a glaucoma shunt tube might be anticipated, this was not observed in the two patients. The viability of EndoART in eyes with glaucoma implants could imply a significant advantage over DMEK due to the stress exerted on endothelial cells by these implants and the shortened associated expected life span of DMEK grafts in eyes with a complex history of glaucoma. The absence of complications in these cases is promising for EndoART as a surgical solution for patients with endothelial failure after glaucoma surgery. Despite successful implantation in this series in an eye with a glaucoma tube, it is still unclear whether glaucoma implants increase the risk of dehiscence. However, the immediate implant adhesion in Patient 7 shows that it is possible to achieve seamless success EndoART implantation despite prior glaucoma shunt implantation. Implantation in larger subsets of eyes with different history profiles will undoubtedly allow for more focused conclusions to be drawn regarding the optimal area of application for this implant [11].

While anterior chamber depth may have an effect on implant behavior, the measurements obtained in this case series are inconclusive in this regard. Further factors that likely affect adhesion behavior include postoperative IOP stability, compliance in patient positioning, and intraocular inflammation. Adjustments in surgical technique including additional sutures may be able to offset conditions that impede adhesion.

The clinical significance of re-bubbling after EndoART differs from re-bubbling after DMEK, since factors affecting graft quality as well as concerns regarding donor cell loss are nullified with this product. Still, multiple re-bubbling procedures are a burden on the patient and the medical system. Some patients might not tolerate multiple interventions with repeated detachments of the artificial endothelial layer.

Early results after EndoART implantation have shown excellent results with the rapid resolution of corneal edema and pain reduction after implantation. Improvement in visual acuity, however, was only slight due to ocular comorbidities in the eyes chosen for implantation [2,4,14]. EndoART therefore appears to offer a generally effective alternative to DMEK grafts in eyes with poor prognoses for endothelial graft longevity due to pre-existing comorbidities such as uveitis or glaucoma.

While increases in visual acuity were modest, this aspect is not necessarily the defining measure of success in the patient cohort that is currently eligible for EndoART implantation, as the potential visual acuity of the eyes that received the implant was restricted by other ocular conditions. However, improvements in photophobia, recurrent infections and pain related to corneal edema associated with endothelial failure are highly relevant for overall quality of life.

Currently, EndoART appears to be a viable alternative to DMEK, particularly for patients with poor prognoses regarding expected graft survival. Being able to provide these patients with an implant that alleviates pain and enables visual rehabilitation as allowed by the visual acuity potential of their eye, which will remain functional despite difficult intraocular conditions, implies a significant improvement in patient care. At the same time, having a viable solution for eyes with poor prognosis for biological grafts frees DMEK grafts for eyes with a higher likelihood of longer-term graft survival. Overall, considering the global shortage in corneal grafts, the supplementation of biological grafts with substitutional implants in suitable cases represents a major advance in the care of all patients with endothelial failure.

## Figures and Tables

**Table 1:** Summary of patient characteristics pre- and postoperatively receiving an EndoART implant.

Patient	Comorbidities and Previous Surgeries	Pachy Min PreOP	AvgK Posterior Cornea (D)	Max. Difference in Posterior Corneal Elevation (µm)	ACD PreOP (mm)	Glaucoma Implant PreOP	Trabeculectomy PreOP	Re-Bubbling	Days Until Adhesion	Pachy Min. PostOP (3 Months)
1	Uveitis, 4 × DMEK, glaucoma, CME	586	−6.68	46	3.09	No	No	0	0	371
2	Trauma 2005, glaucoma, artificial iris, VE + MP, CME, DMEK	533	−6.8	159	3.81	No	Yes	0	0	482
3	Chemical burn, LSCD, DMEK	1034	−6.79	204	3.93	No	No	0	0	550
4	3 × DMEK, CME, glaucoma	559	−6.69	76	3.5	No	No	1	14	423
5	2 × PKP, corneal neovascularization, PAS, glaucoma	660	−6.27	106	2.29	No	No	0	0	409
6	cong. glaucoma, 2 × PKP, CDL, XEN, retinal detachment	731	−6.43	407	5.04	Yes	No	3	never	519
7	Glaucoma, XEN, Ahmed valve, 2 × DMEK, PAS	703	−6.61	105	3.61	Yes	Yes	0	0	372
8	3 × DMEK, glaucoma, uveitis	602	−6.35	129	3.35	No	No	1	7	496
9	St.p. mult. PKP, PAS, corneal NV	741	−6.55	414	2.84	No	Yes	2	never	499
10	St.p. DMEK, AC IOL	575	−6.1	76	3.46	No	No	0	0	445
11	Chemical burn, glaucoma	825	−7.2	92	3.17	No	ZDL, multiple	0	0	-

## Data Availability

Data can be made available upon reasonable request by the corresponding author (ruth.donner@meduniwien.ac.at).
